# Attention Components and Spelling Accuracy: Which Connections Matter?

**DOI:** 10.3390/children8070539

**Published:** 2021-06-24

**Authors:** Lucia Bigozzi, Chiara Malagoli, Chiara Pecini, Sara Pezzica, Claudio Vezzani, Giulia Vettori

**Affiliations:** 1 Department of Education, Languages, Intercultures, Literatures and Psychology (FORLIPSI), University of Florence, Via di San Salvi 12, 50135 Firenze, Italy; lucia.bigozzi@unifi.it (L.B.); chiara.pecini@unifi.it (C.P.); claudio.vezzani@gmail.com (C.V.); giulia.vettori@unifi.it (G.V.); 2 Italian Association for Attention Deficits and Hyperactivity Disorder (A.I.D.A.I), Viale F. Redi 127, 50125 Firenze, Italy; spezzic@yahoo.it

**Keywords:** attention, writing, transparent orthography, working memory, primary school

## Abstract

Attention and working memory are cross-domain functions that regulate both behavioural and learning processes. Few longitudinal studies have focused on the impact of these cognitive resources on spelling skills in the early phase of learning to write. This longitudinal study investigates the contributions of attention and working memory processes to spelling accuracy and handwriting speed in 112 primary school children (2nd, 3rd, and 4th grade; age range: 7.6–9.4 years) learning to write in the Italian transparent orthography. Standardised batteries were used to assess their attention and working memory skills, as well as their spelling. Homophone and non-homophone errors were measured, as they may involve different attentional and working memory processes. The results showed that, for 2nd grade children, selective attention shifting, planning, and inhibition predicted non-homophone errors, whereas sequential working memory predicted homophone errors and writing speed was explained by planning and selective attention. In 3rd grade, only homophone errors were predicted by planning and inhibition. No significant relationships were found in 4th grade, nor in the transition across grades. Dynamic and diversified roles of attentional and working memory processes in predicting different writing skills in early primary school years emerged, with a gradual decrease in the attention–writing relationship with age.

## 1. Introduction

The development of behavioural and learning skills is underpinned by common general domain processes, such as attention and working memory [1]. Attention is an important cognitive domain with a multifaceted nature, which has been proved to be strongly implied in school learning [2]. The efficiency of multiple attention processes transversally affects children’s learning of school subjects in daily-life contexts which are extremely rich in stimuli, such as at school [3,4]. Attentional skills and the capacity to proficiently control them undergo a prolonged development, up to adolescence [5]. Attentional performance increases following a continuum [6], and diverse attentional processes develop from early primary school years to about 9 years of age, a developmental stage during which they reach a more solid and defined factorial structure [7]. Several studies have investigated the existing link between attention and literacy skills [8], documenting an important connection between attention and writing accuracy in different populations with both typical and atypical development [9,10,11]. A high number of children with ADHD commit more spelling errors, in comparison with typically developed peers [12]. The presence of writing difficulties in children with ADHD has been reported in several studies, which also documented the stability of such an association [13], as well as connections between learning difficulties, hyperactive behaviours, and emotional problems in early elementary school [14]. Studies focused on the early stages of formal writing learning could, indeed, enrich our understanding of the genesis of later writing difficulties, informing us of the contributions of several cognitive skills, namely attention, executive functions, and working memory, which may represent distal factors contributing to the automatisation of writing [15].

This study aimed to investigate the patterns of relationships between attention and working memory on spelling skills using a longitudinal research design. This research step will contribute to clarifying whether attention and working memory are involved in supporting spelling skills across primary school years.

### 1.1. Attention and Working Memory

Attention is considered a multicomponent system involving different processes, which is possible to distinguish at functional and anatomical levels [16,17]. Naglieri [18] has defined several specific attentive dimensions, in particular *focused attention*, which refers to concentration directed toward a particular activity/stimulus; *selective attention*, connected to the inhibition of responses to distracting stimuli and interference management; and *sustained attention*, connected to the maintenance of concentration over time, which can be influenced by the different amount of effort required. Among these three components, selective attention is the mechanism that determines what we see and act upon; it has been considered a result of the interaction between an observer’s intentions and goals (current selection goals) and the physical properties of the sensorial environment (salience of the objects). In this perspective, Naglieri and Das [19,20] have theorised the PASS theory (Planning, Attention, Simultaneous, and Successive), which postulates that selective attention and the shifting of the attentional focus work as interconnected systems with more controlled processes, namely planning and inhibition. In accordance with this conceptualisation, specific cognitive processes (e.g., inhibition and working memory) can be viewed as cognitive subcomponents of a single supervisory attentional mechanism, typically including inhibition and the ability to update information maintained in working memory (WM) [21]. The cognitive skills necessary to support this capacity comprehend the attentional control of short-term memory, which supports the ability to integrate, process, dispose and retrieve specific information. In this perspective, the main feature of the active attention system refers to the ability to control attention and preserve a limited quantity of information actively available, specifically in the presence of interference [22]. Similarly, WM interlaces with attentional skills—specifically, with selective attention—comprehending mnemonic components of attention [23].

In this regard, studies already present in the literature have documented the predictive role of attention regulation [24], inhibition [25], and working memory [26,27] processes on writing in children attending primary school. A relation has been also confirmed in atypical samples of children [28,29,30], whose automatic and controlled components of attention may be impaired [31]. Several studies, in fact, have shown that attention deficits are associated with difficulties in writing quality, the number of genre elements, vocabulary, orthographic coding, and handwriting [32]; for further detail, see the review by Graham et al. [33]. Attentional executive skills (e.g., planning, translating, reviewing, and revising) are crucial for writing development, as they allow for the self-regulation of textual writing [34].

### 1.2. The Development of Spelling Competence and the Relation with Attention, Working Memory

The early years of primary school are a significant developmental phase for spelling competence. The ability to write words quickly and accurately is crucial for the development of proficient writing and reading skills and has been associated with successful school outcomes [35]. Spelling, however, is not simple to master.

The dual route model led us to consider spelling and reading as similar processes [36,37]. The literature has recognised that they depend on the same knowledge of the alphabetical system and of the spellings of specific words, but dissociations have also been observed [38]. The dual-route cascaded model, initially proposed to describe acquired dysgraphia in adults [39] and to systematise adult cognitive mechanisms for single-word reading [40,41], has been successfully applied to explain how spelling can be achieved by two main different processes or routes: “*sub-lexical*” and “*lexical*” [42]. According to our knowledge, several authors have described spelling as following dual-route models [43,44,45,46,47]. They predicted that spelling relies on efficiencies of both phonological and lexical processes, which writers need to progressively master. The lexical procedure consists of accessing word-specific memory, while the sub-lexical procedure relies on exploiting regularities in phoneme–grapheme correspondences. In a transparent orthography, such as Italian, children in the early phase of spelling development (and, generally, when they find an unfamiliar word) rely on the “*phonology to orthography conversion system,*” which is the “*sub-lexical*” route to spelling. Children spelling familiar words rely, instead, on the “*lexical*” route, which consists of retrieving the stored spelling of the target word from the “*orthographic lexicon,*” being connected to phonological and semantic information [35].

Some research on typical development has documented that early spelling processes are constrained by attention and executive processes [48,49] and, in particular, that there exists a connection between inattentive behaviour and specific types of errors; for example, in the case of homophone errors [50]. However, longitudinal studies on the relationships between attention, working memory, and spelling skills in the early phase of learning to write in primary school are still scarce. Such an investigation would be particularly interesting in early phases of learning to write a regular orthography; that is, when children are passing from the use of the sub-lexical to the lexical route. In the early phase of learning to spell words, attentional processes are useful to select relevant information, as well as to store and process words in the graphemic buffer and in the orthographic lexicon [51]. As reported by Cloutman et al. [52] (p. 249), “The graphemic buffer is a working memory component of the spelling system that temporarily holds the sequence of graphemes (abstract letters) during production of letter shapes for written spelling or letter names for oral spelling”. Attentional processes allow for efficient access to the graphemic buffer, making writing more accurate [53]. In the face of the well-documented role of verbal working memory skills [54], the predictive contribution of visuo-spatial working memory skills is currently scarcely explored across language systems. The correct orthographic representation of letters, words, and sequences of words with the orthographic representation also relies on visuo-spatial working memory skills [55], as has already been documented in the early phase of writing development [56]. Prior research has identified a relationship between non-homophone (e.g., accents and doublings) and attention [12]. *Sub-lexical route processing* largely involves children’s attention resources in the initial phonological analysis and segmentation of word units; then, during the appropriate conversion of each phonological to orthographic unit, recalling the correct representation and inhibiting the interference of similar phonologically plausible units; finally, assembling each orthographic unit into a correct sequence of letters. The automaticity of the spelling process may suffer from the great demand for inhibitory control and shifting the focus of attention [21], resulting in different orthographic errors. A previous study has shown the relationship between the production of homophone errors and low levels of sustained attention [50]. Those results, obtained in children writing in a Chinese language, led us to reflect on the relationship between homophone errors and attention resources in an alphabetical orthographic system. Considering that homophone words have several spelling options for a shared phonological representation [57], several processes of cognitive control (e.g., selective attention and inhibition) are required [58]. When children are performing a prolonged writing task, it is possible to expect that sustained attentional processes might also play an important role when retrieving the appropriate graphemes for the target word when orthographic conventions are still not completely automatised. Sustained attention processes, in fact, might help to maintain focus on the task, to avoid distractions, and overcome interferences or cognitive conflict [59]. The further involvement of attention and working memory processes has been considered in relation to basic writing skills, such as handwriting speed. Given that handwriting speed is the result of a complex interplay between cognitive, motor component of writing, visuo-spatial, and oculomotor coordination processes [60], it is possible to expect a contribution of attention and working memory efficiency processes to influence children’s handwriting speed performance. Indeed, the association between handwriting difficulties and attention deficits has been highlighted in the literature [61].

Given this evidence, it seems crucial to determine the predictive impact of attentional and working memory processes on writing in an early phase of writing development, including both components of writing processes (i.e., spelling accuracy and handwriting speed). The existing literature, in fact, includes a number of studies assessing specific cognitive aspects connected to writing, such as phonological working memory [62], whereas less is known about the role of attentional-related processes in supporting the development of writing skills.

### 1.3. The Development of Spelling Competence in a Transparent Language System

Spelling processes may be challenged by the specific characteristics of the orthographic language systems. The Italian orthography system is much more transparent than other language systems, such as English or French [63], due to the phoneme–grapheme correspondence rules. The 48 Italian phonemes distinguish 21 single consonants, 16 double consonants, seven pure vowels, and four diphthongs. For most of the consonants and the four diphthongs, there is only one spelling. Many familiar words in the Italian language, such as *mela* [ˈmeːla] (“apple”), are composed of predictable phoneme–grapheme correspondences [64]. Spelling is achieved through shared conventional sound–sign correspondence rules and relies on sensitivity to word sounds [65]. The sound recognition of phonemes in words has an important role in writing orthographic regularities, such as when facing consonant letter doubling or the marking of words with accents. Nevertheless, in Italian—as in most regular orthographies—there is a certain degree of ambiguity in oral-to-written direction, and there are several instances of unpredictable spelling; for instance, the phonemic group [kw] may be transcribed by the orthographic sequences QU or CU [66,67,68].

The correct writing of a homophone word is driven by defined spelling rules [69]. In Italy, primary school children are exposed to spelling instruction from the end of the first year and should be able to fluently retrieve specific orthographic features during spelling production by the third year of primary school [35,70,71]. For example, the phoneme/k/, before the vowel <u>, has two different spellings: in the word *cuore* [‘‘kwͻre] (“heart”), it is spelled <c>, while in the word quadro [‘kwadro] (“painting”), it is spelled <q>. Therefore, children writing *quore* and *cuadro* are committing spelling errors [67,72,73,74]. With regard to consonant letter doubling, in the specific case of the Italian language, the single pronunciation of a consonant phoneme is always written with a single consonant grapheme, while the double pronunciation of a consonant phoneme is almost always written with doubled consonant graphemes. For example, in the word “mamma” (“mum”), the initial/m/is pronounced and written single, whereas the central part/mm/is pronounced and written double. Consequently, when a child writes “mama” for “mamma”, he/she commits a spelling error of non-homophone type as, which relying on the sound of the word, it is possible to distinguish the target word “mamma” from the misspelled word “mama”. Besides sensitivity to word sounds, children’s knowledge of the morphology and conventional spelling rules is important when they learn to read and to spell, both in irregular and in regular orthographic language systems [75]. Children might encounter some difficulties in the phoneme-to-grapheme correspondence rules when they need to automatise spelling rules. In these cases, children cannot rely upon their sensitivity to the sound of words; otherwise, they need to know the specific spelling rule to avoid committing such spelling errors. Specific cases are the following: <ch> preceding <i, e>, pronounced/k/and spelled/kk/, except before <e, i>; <ci> preceding <a, o, u>, pronounced/tʃ/; <gh> preceding <i, e>, pronounced/g, gg/; <gi> preceding <a, o, u>, pronounced/ʤ/; <gl> before <i>, pronounced/ʎ, ʎ ʎ/; <gn> pronounced/ɲ, ɲ go/; <sc> preceding <i, e>, pronounced/ʃ, ʃʃ/; <cch> spelled/kk/only before <e, i>; finally, <sci> spelled/ʃ, ʃʃ/, except before <e, i>. In the Italian context, there are authoritative assessment instruments [76,77,78]: the classification of [74] adopted the two typologies described above for non-homophone and homophone errors.

### 1.4. This study

We investigated the predictive role of attentional and WM skills on spelling accuracy and handwriting speed, in a transparent orthography language system, among primary school children attending 2nd and 3rd grade, followed longitudinally for one year; thus, children in 2nd grade were followed until 3rd grade, while children that were in 3rd grade were followed until 4th grade. We focused on the early phase of writing acquisition, in order to examine whether and how different attentional and WM components are involved in the process of learning orthography. Exploring the above-mentioned relationships allowed us to advance our knowledge of their independent role or their reciprocal influence over early years of schooling. It will be particularly useful to pursue this research aim in a transparent language system, by analysing both homophone and non-homophone errors in spelling. Considering that the level of orthographic transparency has an impact on spelling development, the results of this study, focused on the Italian language system, will inform other transparent orthographies (e.g., Greek) promoting, at the same time, a better comparison of results across more opaque or transparent language systems. Overall, this study will add knowledge about the role of two basic cognitive processes—attention and working memory—in their linkage with spelling accuracy and handwriting speed among typically developed (TD) children. Knowing the developmental pattern of their relationships may be useful to understand the link between attentional and writing disorders.

#### Aim and Hypothesis

This study had the aim of investigating the predictive role of attention and working memory components on spelling accuracy and handwriting speed in 2nd and 3rd grade children, and one year later in the same children attending the subsequent school grade. Coherently with the existing literature (see, e.g., [70,79]), we expected that attentional and working memory components would contribute to predicting spelling accuracy and handwriting speed in 2nd grade, when these processes involve significant effort. Specifically, we expected focused attention and selective attention, together with planning, inhibition, and working memory, to be predictive of spelling accuracy and handwriting speed. Considering 3rd and 4th grade, we expected a weaker predictive contribution of attention and working memory components as, in 3rd grade, orthographic representations are expected to be consolidated and immediately available (see, e.g., [22,64,80]). It was expected that, from 3rd grade, spelling would be more automated, requiring a reduced amount of attentional control and working memory, in comparison to the first two grades. Working memory remains important over time until adulthood; for example, in cognitively complex tasks such as textual writing and taking notes.

## 2. Materials and Methods

### 2.1. Participants

A total of 112 children (52 males; age range: 7.6–9.4 years) took part in the research. Children were selected from an initial sample of 143, from which 19 participants were excluded from the analysis, given that Italian was not their L1, while 12 children were not considered in the analysis given that they had a previous diagnosis for language or other developmental disorders.

In order to prevent data misinterpretation, two subtests from the Wechsler Intelligence Scale [81,82] were used to exclude children with a lower level of functioning [83]. None of the children registered a score below the normal range; thus, none were excluded from the analysis.

The sample, at Time 1, included 59 TD children (28 males; mean age = 7.4 ± 0.42) attending the 2nd year of primary school and 53 pupils (24 males; mean age = 8.5 ± 0.53) attending 3rd grade. At Time 2, 19 pupils of the first data collection did not participate in the second data collection; thus, during the second evaluation, as a whole 93 children were evaluated again, 59 pupils (28 males; mean age = 8.5 ± 0.71) attending the 3rd year of primary school and 34 pupils (17 males; mean age = 9.5 ± 0.69) attending 4th grade, participated in the study (see, Appendix A). All children attended a school in a middle-class suburb of a large city in central Italy. The tasks and tests were administered to the whole class of children. The assessments were administered after agreement with the school director and by respecting the local privacy rules and the policy of informed consent (Italian Legislative Decree DL-196/2003). The World Medical Association guidelines for conducting research, described in the most recent version of the Declaration of Helsinki, were followed. In addition, the research program was approved by the Department of Psychology (University of Florence) before the constitution of the Ethical Committee (2016).

### 2.2. Procedures

Before the research started, pupils’ parents signed the informed consent module. The research intents were made comprehensible to pupils and their parents. Trained experts in Educational Psychology collected the data in a group session lasting about one hour, and a 40 min individual session. The data collection took place during the final part of the Italian school year in the months of May–June per annum. A longitudinal research design was as follows. During the first collection of data (Time 1), the children were attending 2nd and 3rd grade. After one year, a second collection of data (Time 2) was implemented with the same children who were attending, respectively, the 3rd and 4th grade. Subtests from the Cognitive Assessment System (CAS; matching numbers, planned codes, expressive attention, number detection and receptive attention), the BVS-Corsi battery (Paths and Corsi’s block tapping span task), and the BVSCO battery (text dictation and handwriting speed) were administered during the first data collection. During the second data collection, in order to comply with the school’s request of reducing the participating children’s time schedule, the handwriting speed and working memory tasks were not administered, while the other tests and tasks were administered again.

### 2.3. Selected Tasks

#### 2.3.1. General Cognitive Functioning Task

The Similarities and Block Design subtests from the Wechsler Intelligence Scale (WISC-III) were used as control variables [81,82].

#### 2.3.2. Writing Tasks

##### Writing Accuracy

All children completed a paper and pencil text dictation task from the “Battery for the Evaluation of Writing and Orthographic Competence in Primary School” standardised for the Italian population (BVSCO; [78]). Text dictation allows one to analyse children’s writing skills within an ecological setting provided by the semantic context. Furthermore, previous studies have shown that children’s orthographic skills are stable across writing tasks, even though they may involve different cognitive processes (see, e.g., [73]). According to the primary school grade, the appropriate dictation text was used. Children listened to a recorded text and then had to write the text down. To assess children’s spelling accuracy, we adopted the orthographic error classification by Pinto et al. [74], which distinguishes between “*homophone errors*” and “*non-homophone errors,*” covering the entire variability of orthographic errors. In fact, “*homophone errors*” occur when the pronunciation of the target word is preserved, despite the spelling violation (e.g., “anno” [year] instead of “hanno” [they have]). “*Non-homophone errors*” occur when the pronunciation of the target word is changed as a result of the spelling violation (e.g., “mecrato” instead of “mercato”). All phonemes are possible sources of non-homophone errors, such that spelling errors of this type result in phonetically implausible words [35].

Writing accuracy was given by the number of *homophone* and *non-homophone* orthographic errors, which were counted as many times as the error occurred. The chances of homophonic errors depend on the possibilities presented in the dictation. This is different from measuring spelling accuracy in a spontaneous writing production. In the manual of the instrument, the test–re-test reliability regarding errors ranged between 0.57 and 0.84 BVSCO; [78].

#### 2.3.3. Handwriting Speed

Handwriting speed was measured with a BVSCO [78] subtest requiring the children to write down the syllable “*le*” continuously for one minute (e.g., *lelele[…]*), which was timed by the experimenter. The number of correctly written graphemes was the child’s handwriting speed score. In the manual of the instrument, the test–re-test reliability for speed was equal to 0.83 [78].

#### 2.3.4. Attention tasks

The Cognitive Assessment System (CAS) by Naglieri and Das [19] (Italian version by Taddei and Naglieri [84]) is a multidimensional instrument to assess cognitive processing supported by the PASS (Planning, Attention, Simultaneous, and Successive) theory of intelligence, adjusted for children and adolescents from 5 to 17 years of age. The four processing areas of PASS (Planning, Attention, Successive, and Simultaneous processing) comprise the four scales that form the battery. Children’s PASS profiles are relevant to differential diagnosis and are especially helpful for children with learning disabilities and attention deficits [85]; moreover, the battery is considered to be a good predictor of achievement [86], providing information relevant to intervention and instructional planning [87].

For the aim of the present study, we selected the following tasks from the Planning and Attention scales. The internal reliability coefficients were 0.88 for both Planning and Attention.

##### Planning Subtests

Matching numbers task [19,84] (Planning, Selective Attention)—children were presented with a pre-ordered worksheet displaying numbers presented in rows. The task required the identification of identical pairs of numbers in the same row, by underlining them with a pencil. The children were asked to work as quickly and accurately as possible, while they also needed to remember the target numbers and use an effective strategy to distinguish them from the distractors. The time needed to complete the task and the number of correct responses were recorded.

Planned codes task [19,84] (Planning, Inhibition)—children were presented with a pre-ordered worksheet, displaying empty cells in correspondence to the letters A, B, C, and D. The task required children to translate the presented sequence of letters into a specific sequence of symbols (e.g., OX XX OO), based on the provided example presented in the beginning of the worksheet (e.g., A = OX, B = XX). Codes between worksheets changed, together with the direction of codes (vertical vs. diagonal organisation). Children were required to complete the empty cells with the correct code, based on the example provided, while working quickly and accurately. The time needed to complete the task and the number of correct responses were recorded.

##### Attention Subtests

Expressive attention task [19,84] (Inhibition of automatic responses, Interference control)—this is a traditional Stroop task [88]. Children were presented with three sets of items, again in a pre-ordered worksheet. In the case of children up to 7 years, the stimuli consisted of images representing animals. The task required children to identify big or small animals, even though their graphical representations did not correspond to their real-life dimension. Specifically, in item 1, the animals all had the same dimension; in item 2, their dimension was coherent with reality; in item 3, their dimension was opposite with respect to reality. This last item was used to assess the children’s ability to inhibit the interference given by the dimension of the figures and to identify the real size of the corresponding animal. In the case of children from 8 years of age, as in the most traditional Stroop paradigm, items report words, name of colours (item 4), coloured rectangles (item 5), and words reporting the names of colours printed in different colours (item 6). The children were required to name the colour in which the word was written. The child, in this case, has to inhibit the word’s meaning (which is the name of a colour) and state the colour in which the word was printed, while working as quickly and accurately as possible. Similar to the previous set of items, reserved for smaller children, the last item was used for scoring. The time needed to complete the task and the number of correct responses were recorded.

Number detection task [19,84] (Selective Attention, Shifting Focus)—children were presented with a pre-ordered worksheet displaying rows of numbers, among which they are instructed to find and identify target numbers among a number of distractors, by underlining them, following the provided example, working as quickly and accurately as possible. The time needed to complete the task and the number of correct responses were recorded.

Receptive attention task [19,84] (Focused Attention)—children were presented with a pre-ordered worksheet displaying pairs of figures. In the case of children up to 7 years, the sheet was based on graphical similarity or category while, in the case of children from 8 years up, the sheet was based on pairs of letters, which were graphically identical or that have the same name. The child was required to identify, while working quickly and accurately, pairs of items matching on the basis of the graphic similarity or, subsequently, on the basis of the category (name) to which they belong to, among distracting stimuli. The time needed to complete the task and the number of correct responses were recorded.

For all CAS subtests, the stimuli were always arranged in columns and rows, in matrix-style sheets. For all tasks, both time and accuracy (the raw score concerning accuracy was obtained by subtracting correct responses and incorrect ones) were recorded, and the dependent variables were the computed final scores, which combine speed and accuracy measures following the conversion table provided by the manual.

#### 2.3.5. Visuo-Spatial Working Memory Tasks

Visuo-spatial WM skills were assessed through Paths and Corsi’s block tapping span task (Backward version) extracted from the BVS-Corsi Battery [89].

Paths [89] (sequential-spatial working memory)—this task required the child to follow verbal instructions given by the experimenter (who described a path) and to indicate (by imagining the track, visualising it in their minds) the supposed arrival on a grid provided by the experimenter at the end of the instructions. The level of complexity increased from 2 × 2 to 6 × 6 grids. Accuracy was measured at the highest level a child was tested at, which constituted the dependent variable. The test reliability was 0.85.

Corsi’s block tapping span task (backward version) [89] (Visuo-spatial working memory)—the child had to recall, in reverse order, a clustered sequence of positions indicated by the experimenter on a nine-block board. The experimenter completed the sequences by indicating one block per second, and no time recording was needed. The span (i.e., the longest sequence a child recalled correctly) was measured, which constituted the dependent variable. The internal reliability coefficient was 0.74.

### 2.4. Statistical Analysis

Descriptive statistics (mean, standard deviation, skewness and kurtosis coefficients, and minimum and maximum values) concerning attentive skills, visuo-spatial WM skills, spelling accuracy (non-homophone and homophone errors), and handwriting speed were computed for each grade (see Table 1). The normality assumptions were verified for all variables and, whenever the distribution resulted as not normal, the appropriate monotone increasing transformations were applied [90]. In particular, the specific monotone increasing transformations applied to the data included power (squared or cube), square root, and natural logarithm transformations.

Pearson’s correlation coefficients were computed between all measured variables (non-homophone errors, homophone errors, handwriting speed, attention, and visuo-spatial WM measures), both in the 2nd and 3rd grades, and the presence of significant differences between correlations in 2nd and 3rd grade was also verified.

In accordance with the aim of this study, for the purpose of identifying which attention and working memory variables predicted spelling accuracy and handwriting speed, linear multiple regression analyses were performed for 2nd and 3rd grades. The multiple regression analysis allowed us to partialise the predictive effects of each regressor, considering each of them simultaneously with respect to the set of independent variables. Coherently with the suggestion of Austin and Steyerberg [91], we used a minimum ratio between the number of participants and the number of variables which was equal to 5. In fact, Austin and Steyerberg [91] argued that, when using a minimum of two subjects for each independent variable (SPV), the bias in estimation of regression coefficients is lower than 10%. Furthermore, with a minimum of 2 SPV, the standard errors of the regression coefficients, the confidence intervals, and the adjusted R^2^ were accurately estimated [91]. For each of the analyses implemented, attention and planning skills, as well as visuo-spatial working memory skill measures, were included as independent variables and the two-error type classes (non-homophone and homophone) as dependent variables, together with handwriting speed. Each regression was performed independently for each dependent variable. Before implementing the linear multiple regression analyses, to exclude the possible presence of multicollinearity, the statistical coefficient VIF (variance inflation factor) was calculated for all the independent variables [92]. Moreover, for each independent variable included in the regression analysis, the effect-size coefficient partial eta-squared (η^2^) was calculated.

## 3. Results

### 3.1. Descriptive Statistics and Correlations

The analysis of correlations (Table 2) showed a consistent pattern of correlations among all attention tasks, whereas visuo-spatial working memory tasks were less associated with attention variables.

With regard to the analysis of correlations in 2nd grade (first data collection), non-homophone errors were negatively associated with planned codes, number detection, and Corsi’s backward span score; homophone errors correlated with matching numbers, number detection, and Corsi’s backward span; handwriting speed correlated with the matching number, planned code, number detection, and receptive attention tasks. Conversely, in 3rd grade (first data collection), the attention and visuo-spatial WM skills variables were not significantly associated with non-homophone errors; homophone errors emerged as negatively associated with scores of the Path test, while handwriting speed emerged as positively associated with the Expressive Attention and Planned Codes tasks (Table 3).

The analysis performed to test the existence of significant differences in correlations in 2nd and 3rd grade showed a significant difference in the correlation between non-homophone errors and Corsi’s backward span measure (z = −2.31, *p* = 0.011) and in the correlation between handwriting speed and matching number tasks (z = 2.50, *p* = 0.006).

Collinearity tests indicated the absence of multicollinearity, as Tolerance and VIF values ranged between 0.54–0.97 and 1.21–1.85, respectively.

### 3.2. Regression Analysis

The results of the linear regression analyses for the first data collection are reported in Table 4. In 2nd grade (1st data collection), non-homophone errors in spelling were negatively predicted by accuracy of Number detection (*t* = −3.47, *p* < 0.001, *η*^2^ *=* 0.161), Planned codes task (*t* = −2.10, *p* < 0.05, *η*^2^ *=* 0.066), Receptive attention task (*t* = −2.93, *p* < 0.01, *η*^2^ *=* 0.120), and Path test (*t* = −2.72, *p* < 0.01, *η*^2^ *=* 0.105), while homophone errors were also negatively predicted by Path test scores (*t* = −2.07, *p* < 0.05, *η*^2^ *=* 0.063) (see Table 4).

With regard to the contribution of the attention and visuo-spatial WM variables, measured in the 2nd grade children (first data collection), to handwriting speed, accuracy in the matching numbers task (*t* = 2.28, *p* < 0.05, *η*^2^ *=* 0.076) was the only statistically significant regressor (see Table 4).

With regard to the analysis concerning spelling skills in 3rd grade children during the first data collection, accuracy in the Planned codes task (*t* = −2.65, *p* < 0.05, *η*^2^ *=* 0.150) was the only significant regressor of homophone errors. No regressor resulted as a significant predictor of non-homophone errors or handwriting speed (see Table 4).

In the second step, taking into account the results of the first data collection, step-wise regression analyses were performed on the spelling performance of the second data collection. In no case, excluding performance at first data collection, were predictors of performance at second data collection found.

Attention and visuo-spatial WM variables measured in 2nd grade children during the first data collection were not a significant predictor of spelling accuracy measured in 3rd grade during the second data collection.

Equally, spelling accuracy variables assessed in 3rd grade children during the first data collection did not predict any of the attention or WM variables measured in 4th grade (2nd data collection).

## 4. Discussion

This study investigated the roles that different attentional components and working memory play in the process of learning orthography in primary school children attending 2nd or 3rd grade who were followed longitudinally for one year. Specifically, the results of this study contributed to expanding our understanding about the predictive weight of attentional and visuo-spatial WM processes for spelling accuracy in a text dictation task. An innovative contribution of the study was the insight that the predictive role of attentional and working memory processes change, when considering the impact on non-homophone and homophone errors across early primary school years in children belonging to a transparent orthographic language system, such as the Italian one.

Considering 2nd graders, the findings showed that several attentional components and visuo-spatial working memory skills differently predicted non-homophone and homophone errors in text dictation.

Non-homophone errors are committed when writers produce a phonologically implausible spelling of the target word. To correctly transcode the heard word’s sound into the corresponding orthographic sign, children rely on the auditory–verbal channel and the conventional signs and rules of their language. Specifically, in the early years of primary school, children strongly adopt sub-lexical route processing, in order to resolve the phonology–orthography conversion task required when learning to spell [28,93]. The non-automatised use of the sub-lexical route requires paying selective attention to the phoneme–grapheme conversion rules, updating the phonological–orthographic representation, inhibiting similar word sounds, and sustaining attention and speed of processing during fast writing. Thus, during the first years of writing acquisition several attentive and working memory components, such as selective attention, shifting focus, planning, inhibition, focused attention, and sequential-spatial working memory, are needed to avoid non-homophone errors.

Considering the homophone errors committed by 2nd graders, visuo-spatial WM skills, rather than attentional skills, played a significant role. This result adds new insights to the literature, documenting how an increased efficiency in working memory skills is connected to better results in tasks that require higher spelling skills [94]. Indeed, homophone errors arise from a violation of the correct transcription at the orthographic level, while plausibility is maintained at the phonetic level. In order to avoid homophone errors, one needs to utilise lexical route processing. During the primary school years, a child’s mental lexicon increases, including information on word meanings, pronunciation, phonological and orthographic representations, and semantic and orthographic neighbours, known as the graphemic buffer [95]. Working memory is necessary for avoiding homophone errors, by maintaining and updating the active state of the orthographic representation while it is inserted in the specific semantic and orthographic context of the phrase; for example, /lago/(lake) vs. /l’ago/(does) depends on the meaning of the phrase. Specifically, the visuo-spatial component of working memory is needed to choose the correct orthographic representation when it is not sufficient to rely on the auditory–verbal channel to produce the appropriate graphemes.

Recapping the results from 2nd graders, during the first phases of writing acquisition, all attentional components are required in order to avoid non-homophone errors, while visuo-spatial working memory affects both non-homophone and homophone errors.

Considering 3rd graders, no attentional and working memory processes predicted non-homophone errors while, for homophone errors, only the predictive role of performance in the planned codes task, which involves both planning and inhibition, was found. This result suggests that 3rd graders require more selective and controlled attentional mechanisms, in order to avoid homophone errors, even if they show more automatised writing skills than in earlier years of school. Children’s planning and inhibition skills, which are greatly involved in the early phase of spelling acquisition, continue to be involved until the 3rd grade of primary school. It can be hypothesised that, from third grade, in order to choose and access the appropriate representation stored in the orthographic lexicon, a child needs to use planning to anticipate the choice of the correct orthographic form, rather than the sequential step-by-step analytic procedure of phonology-to-orthography conversion rules [70]. Moreover, accessing the correct orthographic representation implies inhibiting wrong—but phonologically plausible—orthographic representations that represent distractors; for example, as the case of the above-mentioned “cuore” [kwore] (heart) vs. “quore” or “l’ago” (needle) vs. “lago” (lake) [|laːgo]. Nevertheless, the role of these skills was found only in the planned codes task, a complex task requiring both planning and inhibition simultaneously, but not in the other attentional and working memory measures.

The progressive decreasing role of attention and working memory in the orthographic task, suggested by the analysis conducted in 2nd and 3rd grades separately, was also confirmed by the longitudinal exploration of relationships between attention and visuo-spatial WM, on one hand, and spelling skills, on the other hand. Attentional and WM skills measured in the first wave of data collection, for 2nd and 3rd graders, did not predict spelling accuracy measured one year later (in 3rd and 4th grade, respectively).

The results were coherent with the hypothesis that the several attention processes needed in the first phases of learning the writing task, allowing children to handle monitoring of the conversion rules and construction of the orthographic lexicon, do not longitudinally predict the automatisation of the orthographic access in later phases [96].

Considering handwriting speed, a similar relationship with attentional processes emerged, although it was weaker in comparison to the orthographic acquisition.

In 2nd grade, controlled attentional components, such as planning and inhibition in the matching number test, predicted handwriting speed. In fact, children who were able to evaluate and select a better sequence of thoughts/actions to achieve the desired goal also seemed to show higher handwriting speed, promoting and supporting greater fluency (in terms of syllables per minute in the execution of the grapho-motor act). This finding supports previous studies showing that planning is the best predictor of handwriting legibility [93,97], and that planning, translating, and reviewing all correlate with handwriting proficiency [98].

Overall, the results of the present study provide interesting suggestions. First, they suggest that attention and visuo-spatial WM resources are highly involved in the spelling task in beginning writers while, as the writing process becomes more automatic, the cognitive demand remains high, in order to avoid committing homophone errors, whereas it is freed up for non-homophone errors. The persistent predictive role of attention in homophone errors may be due to the fact that the orthographic adequacy for homophone letter strings is linked to the application of conventionalised rules of orthographic coding, which must be acquired and automatised.

As the study was conducted in Italian, it contributes to enriching the scarce literature on the attentional and memory processes underpinning writing acquisition in languages with regular orthography. Furthermore, focusing on the initial phase of primary school, a stage of development rich in changes in the cognitive domain (see, e.g., [94,99]), allowed us to draw implications for the education and clinical setting.

In the school system, in fact, the habit of stressing the importance of “paying attention” and “focusing” on a specific task, such as spelling, is frequent, but without working explicitly on the processes that promote the underlying attentional skills. The results of this study highlight that the attention teachers request from their students is useful and functional in the initial phase of spelling acquisition, as well as when the learning process is in action. Attention and visuo-spatial WM skills exert a supportive role when the writing process is not yet fully automatised. In this perspective, acknowledging the role that these components could play in predicting specific learning outcomes may also become an important tool for teachers. Teachers may support the learning process through specific activities, such as planning activities (see, e.g., [100]), visuo-spatial WM games (see e.g., [101]), or the management of interferences (see, e.g., [102]). In fact, neuroscientific research has informed us about the plasticity of these crucial attentive control processes, which are very sensitive to individual differences and may be influenced by environmental stimuli [103].

Although this study investigated important components of attention and visuo-spatial WM skills and their influence in explaining writing, a few limitations of this work warrant mentioning. One important limitation of this study is that we did not examine the role of phonological WM, which can be considered a proximal predictor of spelling [15]. In addition, the features of the words (e.g., frequency and length) used in the writing task were not controlled, which could account for errors and speed. Specifically, the analysis of frequency and number of neighbours of homophone words might provide valuable information about the role of inhibition in homophone error rate, a relation that, in the present study, emerged in 3rd graders. A further limitation is that it was not possible to administer handwriting speed and visuo-spatial WM tasks during the second data collection; in addition, the samples were limited in size.

Furthermore, although the present study may provide some insights on the different roles of attention and WM in the first stages of writing, future studies are still needed. First, the pattern of relationships that emerged in this study should be confirmed through further longitudinal research adopting a longer temporal window and more complex writing tasks. For example, it could be useful to examine the predictive effect of attention and visuo-spatial WM on textual production in older children. Such a research step might help to expand our knowledge regarding whether and how attention and visuo-spatial WM components might come into play in different writing tasks at different ages. Further, our results need to be replicated in experimental conditions evaluating the ecological characteristics of the school environment [104]; for example, the level of classroom noise and chatter (see, e.g., [105]) may prove to be useful for our understanding of the roles of attention and visuo-spatial WM in writing tasks.

## Figures and Tables

**Table 1:** Descriptive statistics of all measures about writing, attention, and visuo-spatial working memory skills in 2nd and 3rd grades (T_1_) and in the next year (T_2_): minimum, maximum, mean, standard deviation, skewness, and kurtosis.

	**Time 1 (2nd grade *N* = 59)**						**Time 1 (3rd grade *N* = 53)**					
	Min	Max	M	SD	Skewness	Kurtosis	Min	Max	M	SD	Skewness	Kurtosis
Non-homophone errors (*)	0	21	5.8	4.80	1.03	0.48	0	31	10.34	7.35	0.88	0.15
Homophone errors (*)	0	7	1.59	1.51	1.12	1.32	0	6	1.24	1.50	1.34	1.29
Handwriting Speed	18	78	53.36	11.76	−0.17	0.18	44	94	68.61	10.96	0.45	0.54
**Attention**												
Matching numbers	4	19	10.86	2.80	0.08	0.13	5	17	10.57	2.32	0.21	0.48
Planned codes	3	15	10.01	2.55	−0.71	0.35	1	18	10.1	3.23	0.12	−0.12
Expressive attention	1	13	7.65	2.95	0.14	−0.71	4	18	9.45	3.61	0.56	−0.51
Number detection (**)	2	19	11.97	3.41	−0.35	0.64	3	16	11.41	2.66	−0.81	1.35
Receptive attention	3	19	11.13	3.32	0.12	−0.26	4	16	10.4	2.42	−0.17	−0.13
**Working Memory**												
Paths	0	29	10.16	6.17	0.73	0.50	2	29	13.28	6.94	0.48	0.06
Corsi’s backward span	2	6	3.86	0.90	0.28	−0.47	2	7	4.52	1.33	0.11	0.15
	**Time 2 (3rd grade *N* = 59)**						**Time 2 (4th grade *N* = 34)**					
**Writing**												
Non-homophone errors (*)	0	33	7.66	8.35	1.43	1.44	0	19	3.83	3.97	1.75	3.75
Homophone errors (*)	0	10	2.39	2.88	1.50	1.17	0	6	0.5	1.25	3.13	10.27
Handwriting Speed												
Matching numbers	6	15	10.32	2.13	0.04	−0.48	6	14	10.31	2.17	−0.28	−0.93
Planned codes	3	15	8.68	2.79	−0.41	−0.03	5	19	11.13	3.50	0.40	−0.52
Expressive attention	4	16	9.64	3.27	0.37	−1.08	1	16	9.38	3.43	0.21	−0.31
Number detection (**)	8	15	11.1	1.95	0.25	−0.99	8	17	11.78	2.62	0.35	−1.03
Receptive attention	5	16	11.32	2.28	−0.54	0.01	5	18	10.96	2.81	−0.13	0.32
**Working Memory**												
Paths												
Corsi’s backward span												

Note. Cognitive functions involved in each test: matching numbers = Planning, Selective Attention; Planned codes = Planning, Inhibition; Expressive attention = Inhibit automatic responses; Number detection = Visual Selective Attention, Shifting Focus; Receptive attention = Focused Attention, interference control; (*) = variable normalised by the application of square root (i.e., a particular increasing monotonic transformation). (**) = variable normalised by the application of square transformation (i.e., a particular increasing monotonic transformation).

**Table 2:** Correlation analyses between attention and visuo-spatial working memory skills variables in 2nd and 3rd grades, first data collection overall: Pearson’s linear correlation coefficient (*N* = 112).

	Matching Numbers	Planned Codes	Expressive Attention	Number Detection	Receptive Attention	Paths	Corsi’s Backward Span
Matching numbers	1	0.40 **	0.33 **	0.41 **	0.36 **	0.07	0.03
Planned codes		1	0.13	0.43 **	0.58 **	0.03	−0.18
Expressive attention			1	0.27 **	0.23 *	0.25 *	0.04
Number detection				1	0.51 **	0.03	−0.02
Receptive attention					1	0.19	−0.03
Paths						1	0.04
Corsi’s backward span							1

*Note*. * *p* < 0.05; ** *p* < 0.01.

**Table 3:** Correlation analyses between all measures regarding spelling accuracy, attention, and visuo-spatial working memory skill variables in 2nd grade (*N* = 59) and 3rd grade (*N* = 53) children (first data collection): Pearson’s linear correlation coefficient.

	Measure	Matching Numbers Task	Planned Codes Task	Expressive Attention	Number Detection Task	Receptive Attention	Paths	Corsi’s Backward Span
				Task		Task		
2nd	Non-homophone errors	−0.12	−0.28 *	0.07	−0.40 **	−0.12	−0.16	−0.37 **
	Homophone errors	−0.28 *	−0.23	0.01	−0.26 *	−0.14	−0.14	−0.30 *
	Handwriting Speed	0.46 **	0.39 **	0.03	0.42 **	0.27 *	0.09	0.12
3rd	Non-homophone errors	0.01	−0.05	0.14	−0.13	0.06	−0.23	0.06
	Homophone errors	−0.04	−0.13	0.21	−0.14	0.01	−0.24 *	−0.21
	Handwriting Speed	0.01	0.26 *	0.01	0.33 **	0.18	0.22	0.01

*Note*. * *p* < 0.05; ** *p* < 0.01.

**Table 4:** Summary of the regression model for the first data collection: regression parameter B, standard error of B (SEB), Student’s t-test (t), *p*-value, and partial eta-squared (Partial *η*^2^).

***Non-Homophone Errors (2nd Grade)*** ***(N =* 59*)***	***Independent Variables (2nd Grade)***	***B***	***SEB***	***t***	***p***	***Partial η*^2^**
*R*^2^*_adj_* = 0.30 ***	Intercept	4.277	0.793	5.392	<0.001	0.316
	Matching numbers task (Planning, Selective attention)	0.036	0.050	0.712	0.479	0.008
	Planned codes task (Planning, Inhibition)	−0.110	0.052	−2.104	0.039	0.066
	Expressive attention task (Inhibit automatic response, interference control)	0.015	0.038	0.393	0.695	0.002
	Number detection task (Selective Attention, shifting focus)	−0.007	0.002	−3.473	0.001	0.161
	Receptive attention task (Focused Attention)	−0.142	0.048	−2.927	0.005	0.120
	Paths (spatial-sequential WM)	−0.343	0.126	−2.719	0.008	0.105
	Corsi’s backward span (Visuo-spatial WM)	−0.210	0.128	−1.640	0.106	0.041
***Homophone Errors (2nd Grade)*** ***(N =* 59*)***	***Independent Variables (2nd Grade)***	***B***	***SEB***	***t***	***p***	***Partial η*^2^**
*R*^2^*_adj_* = 0.11 *	Intercept	2.196	0.377	5.831	<0.001	0.351
	Matching numbers task (Planning, Selective attention)	−0.042	0.024	−1.745	0.086	0.046
	Planned codes task (Planning, Inhibition)	−0.015	0.025	−0.607	0.546	0.006
	Expressive attention task (Inhibit automatic response, interference control)	0.020	0.018	1.130	0.263	0.020
	Number detection task (Selective Attention, shifting focus)	−0.001	0.001	−0.754	0.454	0.009
	Receptive attention task (Focused Attention)	0.014	0.023	0.618	0.539	0.006
	Paths (spatial-sequential WM)	−0.124	0.060	−2.066	0.043	0.063
	Corsi’s backward span (Visuo-spatial WM)	0.031	0.061	0.513	0.610	0.004
***Handwriting Speed (N =* 59*)*** ***(2nd Grade)***	***Independent Variables (2nd Grade)***	***B***	***SEB***	***t***	***p***	***Partial η*^2^**
*R*^2^*_adj_* = 0.21 **	Intercept	123.958	1007.803	0.123	0.903	<0.001
	Matching numbers task (Planning, Selective attention)	145.902	63.985	2.280	0.026	0.076
	Planned codes task (Planning, Inhibition)	103.658	66.683	1.554	0.125	0.037
	Expressive attention task (Inhibit automatic response, interference control)	−9.493	48.211	−0.197	0.845	0.001
	Number detection task (Selective Attention, shifting focus)	3.594	2.411	1.490	0.141	0.034
	Receptive attention task (Focused Attention)	−50.776	61.589	−0.824	0.413	0.011
	Paths (spatial-sequential WM)	−19.113	160.532	−0.119	0.906	<0.001
	Corsi’s backward span (Visuo-spatial WM)	77.940	162.718	0.479	0.634	0.004
***Non-Homophone Errors (3rd Grade)*** ***(N =* 53*)***	***Independent Variables (3nd Grade)***	***B***	***SEB***	***t***	***p***	***Partial η*^2^**
*R*^2^*_adj_* = 0.02	Intercept	4.462	1.318	3.387	0.002	0.223
	Matching numbers task (Planning, Selective attention)	0.048	0.072	0.675	0.504	0.011
	Planned codes task (Planning, Inhibition)	−0.130	0.083	−1.554	0.128	0.057
	Expressive attention task (Inhibit automatic response, interference control)	−0.004	0.062	−0.068	0.946	<0.001
	Number detection task (Selective Attention, shifting focus)	−0.001	0.004	−0.201	0.841	0.001
	Receptive attention task (Focused Attention)	0.010	0.097	0.101	0.920	<0.001
	Paths (spatial-sequential WM)	−0.141	0.327	−0.430	0.669	0.005
	Corsi’s backward span (Visuo-spatial WM)	−0.024	0.159	−0.154	0.879	0.001
***Homophone Errors (3rd Grade)*** ***(N =* 53*)***	***Independent Variables (3rd Grade)***	***B***	***SEB***	***t***	***p***	***Partial η*^2^**
*R*^2^*_adj_* = 0.20 *	Intercept	3.090	0.433	7.128	<0.001	0.560
	Matching numbers task (Planning, Selective attention)	−0.001	0.024	−0.055	0.956	<0.001
	Planned codes task (Planning, Inhibition)	−0.073	0.027	−2.654	0.011	0.150
	Expressive attention task (Inhibit automatic response, interference control)	0.020	0.021	0.970	0.338	0.023
	Number detection task (Selective Attention, shifting focus)	−0.001	0.001	−0.504	0.617	0.006
	Receptive attention task (Focused Attention)	−0.021	0.032	−0.649	0.520	0.010
	Paths (spatial-sequential WM)	−0.196	0.108	−1.823	0.076	0.077
	Corsi’s backward span (Visuo-spatial WM)	−0.009	0.052	−0.173	0.864	0.001
***Handwriting Speed (3rd Grade)*** ***(N =* 53*)***	***Independent Variables (3rd Grade)***	***B***	***SEB***	***t***	***p***	***Partial η*^2^**
*R*^2^*_adj_* = 0.07	Intercept	3287.292	1964.346	1.673	0.102	0.065
	Matching numbers task (Planning, Selective attention)	69.395	106.682	0.650	0.519	0.010
	Planned codes task (Planning, Inhibition)	34.066	124.319	0.274	0.785	0.002
	Expressive attention task (Inhibit automatic response, interference control)	159.808	92.968	1.719	0.093	0.069
	Number detection task (Selective Attention, shifting focus)	−8.388	5.412	−1.550	0.129	0.057
	Receptive attention task (Focused Attention)	189.975	144.315	1.316	0.196	0.042
	Paths (spatial-sequential WM)	−312.355	487.630	−0.641	0.525	0.010
	Corsi’s backward span (Visuo-spatial WM)	−236.092	237.436	−0.994	0.326	0.024

*Note*. * *p* < 0.05; ** *p* < 0.01; *** *p* < 0.001.

## Data Availability

The data presented in this study are available on request from the corresponding author. The data are not publicly available, due to a previous agreement with the schools involved in the study, which ensured that data would be stored at the Scholastic Psychology laboratory at University of Florence without being publicly shared.
